# Comparative Analysis of COVID-19 Outcomes in Type 1 and Type 2 Diabetes: A Three-Year Retrospective Study

**DOI:** 10.3390/medicina60020210

**Published:** 2024-01-25

**Authors:** Flavius Cioca, Romulus Timar, Flavia Ignuta, Adrian Vlad, Felix Bratosin, Ovidiu Rosca, Adelina Maria Jianu, Daniela Rosca, Susa Septimiu-Radu, Sonia-Roxana Burtic, Ariadna Petronela Fildan, Sorina Maria Denisa Laitin

**Affiliations:** 1 Doctoral School, “Victor Babes” University of Medicine and Pharmacy, Eftimie Murgu Square 2, 300041 Timisoara, Romania; flaviuscioca@yahoo.com (F.C.); daniela.rosca@umft.ro (D.R.);; 2 Department of Internal Medicine II, Division of Diabetes, Nutrition and Metabolic Diseases, “Victor Babes” University of Medicine and Pharmacy, Eftimie Murgu Square 2, 300041 Timisoara, Romania; timar.romulus@umft.ro (R.T.);; 3 Centre for Molecular Research in Nephrology and Vascular Disease, Faculty of Medicine, “Victor Babes” University of Medicine and Pharmacy, Eftimie Murgu Square 2, 300041 Timisoara, Romania; 4 Department of Infectious Diseases, “Victor Babes” University of Medicine and Pharmacy Timisoara, 300041 Timisoara, Romania; felix.bratosin@umft.ro (F.B.); ovidiu.rosca@umft.ro (O.R.); 5 Department of Anatomy and Embryology, “Victor Babes” University of Medicine and Pharmacy Timisoara, 300041 Timisoara, Romania; adelina.jianu@umft.ro; 6 Department II, Discipline of Medical Communication, “Victor Babes” University of Medicine and Pharmacy, Eftimie Murgu Square 2, 300041 Timisoara, Romania; 7 Department of Pulmonology, Faculty of Medicine, “Ovidius” University of Constanta, 900470 Constanta, Romania; 8 Discipline of Epidemiology, “Victor Babes” University of Medicine and Pharmacy Timisoara, 300041 Timisoara, Romania; laitin.sorina@umft.ro

**Keywords:** diabetes mellitus, COVID-19, SARS-CoV-2

## Abstract

*Background and Objectives*: This comprehensive retrospective study assesses COVID-19 outcomes in type 1 (T1D) and type 2 diabetes (T2D) patients across three years, focusing on how these outcomes varied with the evolving pandemic and changes in diabetes management. The study aims to determine if COVID-19 outcomes, including severity, intensive care unit (ICU) admission rates, duration of hospitalization, and mortality, are significantly different between these diabetes subtypes. *Materials and Methods*: The study analyzed data from patients admitted to the Victor Babes Hospital for Infectious Diseases and Pulmonology with confirmed COVID-19 and pre-existing diabetes, from the years 2020, 2021, and 2022. *Results*: Among 486 patients (200 without diabetes, 62 with T1D, 224 with T2D), T2D patients showed notably higher severity, with 33.5% experiencing severe cases, compared to 25.8% in T1D. Mortality rates were 11.6% in T2D and 8.1% in T1D. T2D patients had longer hospital stays (11.6 ± 7.0 days) compared to T1D (9.1 ± 5.8 days) and were more likely to require ICU admission (OR: 2.24) and mechanical ventilation (OR: 2.46). Hyperglycemia at admission was significantly higher in the diabetes groups, particularly in T2D (178.3 ± 34.7 mg/dL) compared to T1D (164.8 ± 39.6 mg/dL). *Conclusions*: The study reveals a discernible difference in COVID-19 outcomes between T1D and T2D, with T2D patients having longer hospital admissions, mechanical ventilation necessities, and mortality risks.

## 1. Introduction

Diabetes mellitus, encompassing both type 1 and type 2 variants, represents a significant global health challenge [1]. As of early 2023, the International Diabetes Federation estimated that approximately 537 million adults (20–79 years) were living with diabetes worldwide, with this number projected to rise to 783 million by 2045 [2]. Concurrently, the COVID-19 pandemic, caused by the novel coronavirus SARS-CoV-2, emerged as a defining global health crisis [3]. By early 2023, the World Health Organization (WHO) reported over 500 million confirmed cases globally, with a significant mortality burden [4].

Emerging evidence has consistently highlighted diabetes, particularly poorly controlled diabetes, as a risk factor for severe COVID-19 outcomes [5,6]. Studies have shown that individuals with diabetes are more likely to experience severe symptoms, require hospitalization, and face a higher risk of mortality from COVID-19 compared to those without diabetes [7,8]. The pathophysiological mechanisms underlying this increased risk involve a confluence of impaired immune response, chronic inflammation, and potentially direct pancreatic damage by the virus, which aggravates hyperglycemia [9,10].

The two primary forms of diabetes, type 1 (T1D) and type 2 diabetes (T2D), exhibit distinct pathophysiological characteristics that could differentially impact COVID-19 outcomes [11]. Type 1 diabetes, an autoimmune condition, leads to the destruction of insulin-producing beta cells in the pancreas, resulting in insulin deficiency [12,13]. In contrast, type 2 diabetes primarily involves insulin resistance and relative insulin insufficiency [14,15]. These differences suggest potential variations in the clinical progression and outcomes of COVID-19 in patients with these two types of diabetes.

The COVID-19 pandemic has also influenced diabetes management, with disruptions in healthcare services, changes in physical activity patterns, and psychological stress affecting glycemic control in patients with diabetes [16,17]. This evolving context underscores the importance of reassessing the interplay between diabetes types and COVID-19 outcomes as the pandemic progressed.

The increased risk and mortality from COVID-19 in individuals with diabetes are partly due to compromised immune responses, heightened chronic inflammatory states, and the possibility of direct viral damage to pancreatic cells [18]. These factors exacerbate hyperglycemia and contribute to the deterioration of clinical outcomes. The differential pathophysiology of type 1 and type 2 diabetes further influences these outcomes, necessitating a deeper examination of their respective impacts on the severity of COVID-19. Therefore, this study aims to conduct a comprehensive retrospective analysis of COVID-19 outcomes in patients with type 1 and type 2 diabetes over a three-year period (2020–2022). It is hypothesized that COVID-19 outcomes, including severity, intensive care unit (ICU) hospitalization rates, and mortality, differ significantly between patients with type 1 and type 2 diabetes. Additionally, we aim to explore how these outcomes have evolved over the course of the pandemic, considering the changing dynamics of both COVID-19 and diabetes management. 

## 2. Materials and Methods

### 2.1. Study Design and Participants

This retrospective study evaluated COVID-19 outcomes in patients with type 1 and type 2 diabetes, admitted to the Victor Babes Hospital for Infectious Diseases and Pulmonology from 1 January 2020, to 31 December 2022. Patients aged 18 and above were included, with a pre-existing diagnosis of diabetes (type 1 or type 2 as per American Diabetes Association guidelines) [19], and a confirmed COVID-19 diagnosis, as per the Center for Disease Control criteria [20]. Data were extracted from the database and paper records of the Victor Babes Hospital for Infectious Diseases and Pulmonology tertiary care center.

The study protocol was reviewed and approved by the institutional ethics committee of each participating center. Patient confidentiality was strictly maintained, with data anonymized before analysis. The research strictly followed the ethical guidelines and obtained clearance from the Local Commission of Ethics for Scientific Research, ensuring compliance with the EU GCP Directives 2005/28/EC, ICH guidelines, and the tenets of the Declaration of Helsinki.

### 2.2. Inclusion and Exclusion Criteria

Inclusion criteria comprised the following: (1)—confirmed diagnosis of type 1 or type 2 diabetes, based on ADA criteria (type 1: evidence of autoimmune beta-cell destruction, type 2: evidence of insulin resistance with relative insulin deficiency); (2)—laboratory-confirmed SARS-CoV-2 infection by reverse transcription polymerase chain reaction (RT-PCR); (3)—age 18 years or older at the time of COVID-19 diagnosis; (4)—patients’ consent for analysis of their personal medical records.

Exclusion criteria comprised the following: (1)—gestational diabetes or other forms of diabetes; (2)—patients with evidence of high serum glucose levels but without a clear diagnosis of type 1 or type 2 diabetes; (3)—incomplete medical records or missing key data like HbA1c levels, COVID-19 treatment details, or outcome data; (4)—lack of consent.

### 2.3. Data Collection and Variables

Data were collected from digital and paper records of the patients, and included demographics such as age, gender, place of origin, diabetes type, duration of diabetes, HbA1c levels and laboratory data (with normal ranges according to our laboratory standards), insulin use, comorbidities (cardiovascular disease, neuropathy, kidney disease), Charlson Comorbidity Index (CCI) [21], COVID-19 severity (mild, moderate, severe/critical as per WHO criteria [22]), hospitalization details, patients on dialysis, ICU admissions, mechanical ventilation, and mortality. COVID-19 severity was defined based on symptoms, oxygen requirements, and chest imaging findings. Patients were matched by age, gender, body mass index (BMI), and CCI.

### 2.4. Statistics

Data were analyzed using SPSS Version 26. Descriptive statistics summarized baseline characteristics. Chi-square tests were used to compare proportions of categorical variables, while independent *t*-tests or analysis of variance (ANOVA) were employed for comparing continuous variables’ means and standard deviations. Spearman’s correlation was calculated between the significant study variables. Multivariable logistic regression models identified factors associated with severe COVID-19 outcomes, adjusting for age, gender, BMI, comorbidities, and diabetes duration. Kaplan–Meier curves analyzed time-to-event data, and Cox proportional hazards models evaluated mortality risk.

## 3. Results

### Patients’ Background Analysis

The retrospective analysis of 486 COVID-19 patients, comprising 200 without diabetes, 62 with type 1 diabetes mellitus, and 224 with type 2 diabetes mellitus, revealed no significant differences in age, gender, body mass index (BMI), smoking habits, frequent alcohol use, place of origin, COVID-19 vaccination status, and Charlson Comorbidity Index greater than 2. The mean age was comparable across groups (no diabetes: 55.3 years; T1D: 53.6 years; T2D: 56.4 years; *p* = 0.281). The gender distribution showed a majority of men in each group, but without statistical significance (*p* = 0.586). Similarly, the average BMI across groups showed no significant difference (*p* = 0.234).

However, significant differences emerged in COVID-19 severity. A higher proportion of severe cases were observed in the T2D group (33.5%) compared to the T1D (25.8%) and no diabetes groups (15.0%), with a *p*-value of <0.001. This trend was also reflected in higher rates of ICU admissions and mechanical ventilation in the T2D group (31.7% and 35.3%, respectively) compared to T1D (22.6% and 29.0%) and no diabetes groups (17.0% and 18.0%), indicating a more severe clinical course in T2D patients (ICU admissions *p* = 0.002; mechanical ventilation *p* ≤ 0.001). Mortality rates were also notably higher in the T2D group (11.6%) compared to patients with T1D (8.1%) and those without diabetes (3.5%, *p* = 0.008).

Regarding diabetes-related complications, there were no significant differences in the prevalence of cardiovascular disease, neuropathy, kidney disease, and other complications among T1D and T2D patients. However, hyperglycemia at admission was significantly more prevalent in the T2D group (51.8%) compared to the T1D group (32.3%) with a *p*-value of 0.006. Insulin use was universal among T1D patients (100%) and significantly lower in the T2D group (17.0%), with a *p*-value of <0.001. Dialysis use did not differ significantly between the two groups of patients with diabetes (*p* = 0.377), as presented in Table 1.

Blood glucose levels were significantly higher in the diabetes groups (T1D: 164.8 mg/dL, T2D: 178.3 mg/dL) compared to the no diabetes group (95.1 mg/dL), with a *p*-value of <0.001. HbA1c levels also differed markedly, being within the normal range for the no diabetes group (5.6%) and elevated in the T1D (7.7%) and T2D (8.1%) groups, consistent with their diabetic status (*p* < 0.001).

C-reactive protein levels, indicative of inflammation, were significantly higher in the T1D (27.4 mg/L) and T2D (38.2 mg/L) groups compared to the no diabetes group (15.2 mg/L), suggesting more pronounced inflammatory responses in patients with diabetes (*p* < 0.001). Elevated D-dimer levels, suggestive of coagulation abnormalities, were observed in the diabetes groups (T1D: 2.8 μg/mL, T2D: 2.9 μg/mL) compared to the no diabetes group (1.6 μg/mL, *p* < 0.001).

Oxygen saturation, a key indicator of respiratory function, was lower in T1D (91.7%) and T2D (90.8%) groups compared to the no diabetes group (93.9%), with a statistically significant difference (*p* = 0.037). Creatinine levels, reflecting kidney function, were also significantly higher in patients with diabetes (T1D: 1.2 mg/dL, T2D: 1.3 mg/dL) compared to those without diabetes (0.9 mg/dL, *p* < 0.001). Lymphocyte and white blood cell (WBC) counts, however, did not show significant differences across groups, with lymphocytes and WBC counts being similar, as presented in Table 2.

The analysis of patient outcomes in our study revealed significant differences in the length of hospital stay and the extent of medical interventions among patients with no diabetes, T1D, and type 2 diabetes mellitus. Notably, the length of hospital stay was significantly longer for T2D patients (11.6 days) compared to T1D (9.1 days) and no diabetes groups (7.2 days), with a significant difference (*p* < 0.001), highlighting the increased healthcare burden in T2D patients.

Regarding treatment, the proportion of patients receiving antiviral treatment and steroids was higher in the diabetes groups, particularly in T2D, but these differences were not statistically significant for antiviral treatments (*p* = 0.117). However, the use of steroids was significantly more prevalent in T2D (74.6%) compared to no diabetes group (60.5%, *p* = 0.008), indicating a more aggressive treatment approach in patients with diabetes, especially those with type 2 diabetes.

The requirement for non-invasive ventilation was significantly higher in the T2D group (53.1%) compared to T1D (45.2%) and no diabetes (31.0%) groups (*p* < 0.001), aligning with the observed trend of increased COVID-19 severity in T2D patients. Finally, the discharge status indicated a lower recovery rate in the T2D group (88.4%) compared to the no diabetes (96.5%) and T1D (91.9%) groups, with a statistically significant difference (*p* = 0.008), suggesting the increased risk and complications associated with COVID-19 in patients with type 2 diabetes, as seen in Table 3.

The analysis of COVID-19 outcomes over three years revealed a significant evolution in disease severity, hospitalization length, ICU admissions, and mortality among patients with type 1 and type 2 diabetes mellitus (T1D and T2D). In 2020, both groups had moderate COVID-19 severity, but T2D patients experienced slightly higher rates of severe cases (17.0% vs. 14.5% in T1D) and longer hospital stays (10.8 days for T2D vs. 9.0 days for T1D), alongside higher ICU admissions and mortality rates.

The year 2021 marked a peak in severity, particularly for T2D patients, who exhibited a greater proportion of severe cases (25.0% vs. 20.3% in T1D) and the longest hospital stays (12.4 days for T2D vs. 9.9 days for T1D). ICU admissions and mortality rates were notably higher in T2D during this year, aligning with the observed increase in disease severity.

In 2022, a decline in severe cases was observed, yet T2D patients continued to experience higher severity, longer hospital stays, and elevated ICU admissions and mortality rates compared to T1D patients. These findings highlight the disproportionately higher impact of COVID-19 on T2D patients over the years, with 2021 being the most challenging year, as presented in Table 4.

A significant negative correlation was observed between BMI and COVID-19 severity (ρ = −0.473, *p* < 0.05), suggesting that higher BMI was associated with less severe COVID-19 outcomes. This finding could imply a protective aspect of higher BMI against COVID-19 severity, which contradicts typical health assumptions. Similarly, a significant correlation between BMI and the requirement for mechanical ventilation (ρ = 0.439, *p* < 0.05) was found, indicating that higher BMI might be linked to an increased need for mechanical support due to respiratory complications. Hyperglycemia at admission showed a significant negative correlation with COVID-19 severity (ρ = −0.528, *p* < 0.05) and mortality (ρ = −0.414, *p* < 0.05), highlighting the impact of initial glucose levels on disease progression and survival. This finding underscores the importance of glycemic control in patients with diabetes during COVID-19 infection.

**Figure 1:**
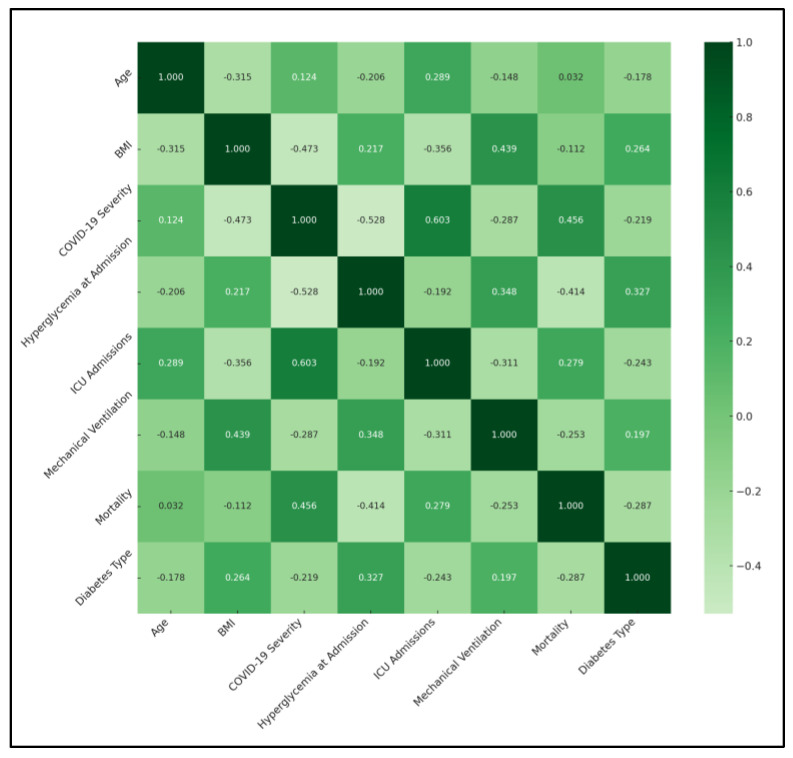
Correlation matrix of COVID-19 outcomes and characteristics.

ICU admissions were significantly correlated with age (ρ = 0.289, *p* < 0.05) and COVID-19 severity (ρ = 0.603, *p* < 0.05), suggesting that older age and higher disease severity were predictive of ICU admission. This outcome aligns with global observations that severe cases of COVID-19 are more likely to require intensive care, particularly among older populations. Notably, the correlation matrix revealed significant relationships involving diabetes type. Diabetes type showed a positive correlation with hyperglycemia at admission (ρ = 0.327), indicating a higher likelihood of elevated blood glucose levels in patients with diabetes, particularly those with type 2 diabetes, as presented in Table 5 and Figure 1.

The ICU admission risk was notably higher in the T2D group at 31.70% compared to 17.00% in the no diabetes group. This significant increase was quantified with an odds ratio (OR) of 2.24, indicating more than double the risk. T2D patients also faced a higher risk of ICU admission than T1D patients (22.60%), with an OR of 1.59, indicating substantially elevated risk of severe COVID-19 complications requiring intensive care among T2D patients.

Similarly, the risk of requiring mechanical ventilation followed a significant trend. For T2D patients, this risk was 35.30%, compared to 18.00% for those with no diabetes. The OR of 2.46 suggested a markedly higher likelihood of needing mechanical respiratory support for T2D patients. The comparison with T1D patients, who had a 29.00% risk, also showed a higher, though less pronounced, risk for T2D individuals.

**Figure 2:**
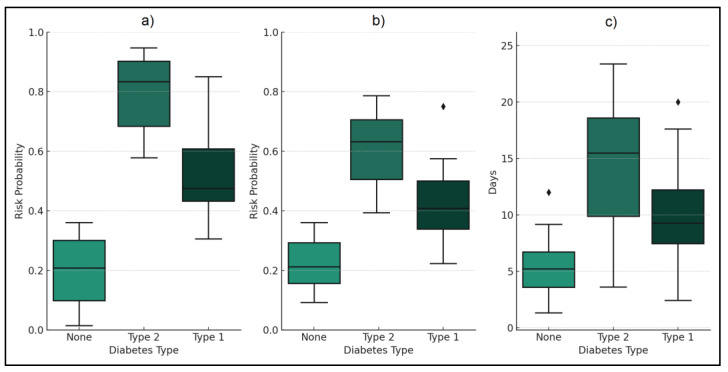
Comparative analysis of COVID-19 clinical outcomes across diabetes and no diabetes groups; (**a**)—ICU admission risk; (**b**)—mechanical ventilation risk; (**c**)—hospitalization duration.

Mortality rates provided further critical insights. T2D patients exhibited a mortality rate of 11.60%, significantly higher than the 3.50% in the no diabetes cohort. The OR of 3.60 underlined the grave impact of T2D on COVID-19 fatality rates. The mortality rate for T1D patients was also higher than for those without diabetes, but the relative increase was lower compared to T2D patients. The duration of hospitalization further mirrored these trends. T2D patients had an average hospital stay of 11.6 days, longer than the 7.2 days observed in the no diabetes group, with an OR of 1.61, as seen in Figure 2 and Table 6.

In the analysis, patients with no diabetes (represented by the light green curve) exhibit the highest survival probability over the observed period. This group’s curve descends most gradually, indicating a lower rate of mortality compared to the diabetes groups. The survival probability for this group remains relatively high throughout the time frame, affirming the less severe impact of COVID-19 on patients without diabetes.

Patients with type 1 diabetes, denoted by the medium sea green curve, show a moderately decreased survival probability compared to the no diabetes group. The curve for T1D descends at a faster rate than that of the no diabetes group, but not as rapidly as the type 2 diabetes group. The log-rank test between T1D and no diabetes yields a *p*-value of 0.068, which is marginally above the typical threshold for statistical significance, suggesting a trend towards but not definitively indicating higher mortality in the T1D group compared to those without diabetes.

**Figure 3:**
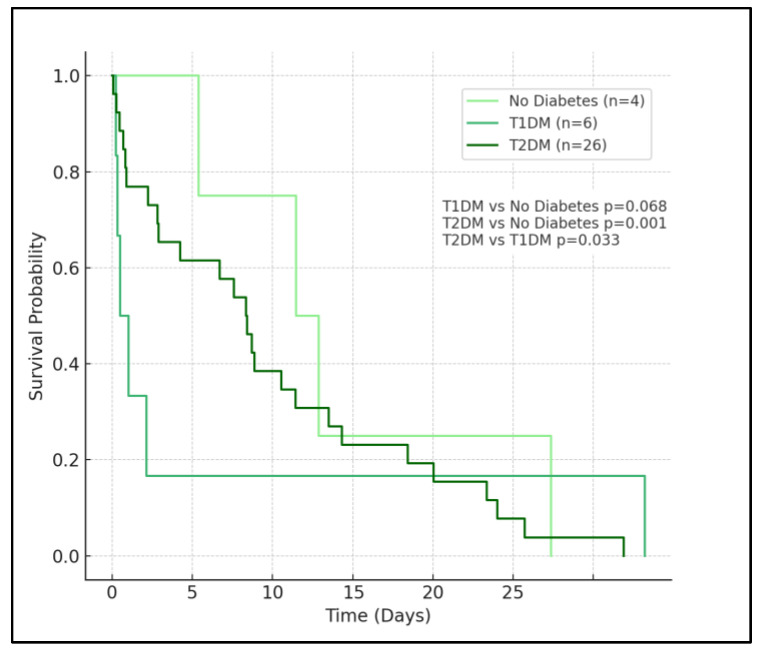
Kaplan–Meier survival analysis curves.

The most notable observation comes from the survival curve for patients with T2D, depicted in dark green. This curve shows a significantly steeper decline, especially in the initial phase, reflecting a higher mortality rate. The rapid descent of the T2D curve underscores the severe impact of type 2 diabetes on COVID-19 outcomes. The log-rank *p*-values corroborate this observation, with a highly significant difference (*p* < 0.001) in survival when compared to the no diabetes group, and a significant difference (*p* = 0.033) when compared to the T1D group, as seen in Figure 3. These results indicate that T2D is a significant risk factor for poorer outcomes in COVID-19 patients. 

## 4. Discussion

The current study observed distinct patterns in COVID-19 severity and outcomes between patients with type 1 and type 2 diabetes mellitus, findings that are critical in understanding the impact of the pandemic on diabetic populations and aligns with emerging global data indicating an increased diabetes risk post-COVID-19 infection. There was a higher rate of severe COVID-19 cases in T2D patients (33.5%) compared to T1D (25.8%). This disparity is reflected in the hospitalization duration, which was longer for T2D (11.6 days) than T1D (9.1 days). These results are consistent with the observed global trend where COVID-19 has been associated with a 66% higher risk of death in patients with diabetes, underscoring the severity of the disease in patients with diabetes [23,24].

Our study also revealed more elevated blood glucose levels at admission in T2D (178.3 mg/dL) compared to T1D (164.8 mg/dL), highlighting the distinct pathophysiological mechanisms of these diabetes types, indicating that SARS-CoV-2 can exacerbate glucose metabolism disorders, as patients without diabetes had a serum glucose level of 95.1 mg/dL. The modest increase in diabetes risk due to COVID-19, as evidenced in our study and global data, suggests an impending rise in global diabetes diagnoses, calling for vigilant monitoring of glucose dysregulation in COVID-19 patients and survivors, especially in countries with high diabetes prevalence [25,26]. The pronounced hyperglycemia observed during stress and infection, a common response in individuals, is notably more severe in patients with type 2 diabetes, as reflected by elevated HbA1c levels. This indicates that these patients often experience poorly controlled diabetes, exacerbating their body’s response to stress and infection.

In our study, T2D patients showed a higher mortality rate (11.6%) compared to T1D (8.1%), and significantly higher than patients with no diabetes (3.5%), which may be indicative of the generally more severe clinical presentation and comorbidities associated with T2D. This aligns with a systematic review that found no significant differences in disease severity but a potentially lower mortality rate in T1D COVID-19 patients [27]. Thus, our study contributes to the limited information on how COVID-19 affects T1D patients. The prevalence of T1D in our COVID-19 cohort was 31% compared to global reports ranging from 0.15% to 28.98%, illustrating the diverse clinical presentations and outcomes in this patient population [28].

In a study covering the initial three waves of the COVID-19 pandemic, researchers observed a significantly higher likelihood of severe COVID-19 outcomes, including hospitalization, intensive care unit admission, and mortality, in individuals with type 2 diabetes, which corroborates our findings that compared three consecutive years of the pandemic [28]. Notably, younger individuals with type 2 diabetes exhibited disproportionately higher odds for these severe outcomes compared to their older counterparts and the general non-diabetic population. For those with type 1 diabetes, the study found a moderate increase in hospitalization rates due to COVID-19, but no significant rise in ICU admissions or mortality was observed. Moreover, the authors emphasized the influence of age and socioeconomic factors in predicting severe COVID-19 outcomes across both types of diabetes.

There was also an elevated risk for severe COVID-19 in individuals under 55 with T2D, probably attributed to fewer competing risks in this younger demographic. The study also shed light on the delicate balance needed in managing type 2 diabetes during the pandemic, especially considering the negative impacts of reduced mobility and the significant role of obesity in worsening COVID-19 outcomes. The findings also underscored the relevance of hypertension and renal disease in the context of SARS-CoV-2 infections [16,29]. In our study the observed negative correlation between BMI and COVID-19 severity appears counterintuitive, given the established health risks associated with higher BMI. However, it echoes emerging evidence suggesting a protective role of higher BMI in the context of COVID-19, also known as the “obesity paradox” phenomenon [30]. One hypothesis for this phenomenon could be that patients with higher BMI receive respiratory support earlier in the course of infection, which may prevent the escalation of severity.

In the context of diabetes management, maintaining controlled glucose levels and avoiding hyperglycemia are crucial for reducing severe COVID-19 risks [31]. Diabetes can lead to inflammatory responses, hypercoagulation, and severe pneumonia, exacerbated by elevated D-dimer and fibrinogen levels. While 2020 data indicated increased risks of ICU care and death post-COVID-19 infection in patients with type 1 diabetes, the current study did not find a significant excess risk of ICU care or death in this group, but mostly in the T2D group [32]. This finding is consistent with other research, despite a longer observation period allowing for more outcomes. Interestingly, the majority of deaths in T1D patients were among those older than 75 years, and after adjusting for comorbidities, there was no increase in odds. This suggests that younger and middle-aged individuals with uncomplicated type 1 diabetes may not face increased risks of severe COVID-19 outcomes compared to their non-diabetic peers [33].

The lesser severity of COVID-19 observed in patients with type 1 diabetes compared to those with type 2 may be partially attributed to the general blood sugar control maintained by insulin therapy. Individuals with T1D often have regimented treatment protocols, including insulin administration, that could result in a more stable glycemic state prior to COVID-19 infection [34]. This contrasts with the varied management effectiveness in T2D, where insulin resistance and other metabolic complications might predispose individuals to worse outcomes when infected. Therefore, the disciplined glycemic control inherent to type 1 diabetes management might confer a relative protective advantage against the severity of COVID-19.

Regarding hospitalization rates, another study found a significant independent increase in the odds of hospitalization for COVID-19 in patients with type 1 diabetes, as opposed to ICU care and death, which was significantly higher in T2D patients, and similar to our study’s findings [35]. This could be attributed to a higher propensity for admitting these patients due to perceived risks. Despite potential reductions in physical hospital visits, there is evidence indicating that glycemic control in T1D patients might not have been significantly affected by the pandemic [36]. The observed low odds of hospitalization and death could also be due to precautionary measures taken by these patients, such as working from home; however, tendencies of increased ICU care and death were observed in the overall analyses.

The elevated white blood cell count in patients with type 2 diabetes, despite a typically suppressed immune response, may reflect a complex interplay of factors during COVID-19 infection. The heightened WBC count could indicate an acute inflammatory response triggered by the virus, countering the expected immunosuppressive state of T2D. While lymphocyte counts remain similar across groups, the increased WBC count in T2D patients could signify a compensatory mechanism or an atypical immune response to infection, as a previous study indicated that WBCs are more significant among patients with diabetes [37].

In our study, several limitations should be acknowledged, such as the retrospective nature of the study at the Victor Babes Hospital for Infectious Diseases and Pulmonology, which inherently limits our ability to establish causal relationships. Secondly, our patient cohort, comprising 486 individuals, may not fully represent the broader population, particularly given the specific healthcare setting and regional demographics. Thirdly, the reliance on existing medical records might have introduced biases related to documentation accuracy and completeness. Furthermore, the exclusion of patients with incomplete records or those lacking consent could have led to selection bias. Finally, the study’s findings may not be generalizable beyond the context of the Romanian healthcare system and the specific period of the COVID-19 pandemic studied.

## 5. Conclusions

Conclusions: This extensive retrospective study provides critical insights into the differential impacts of COVID-19 on patients with type 1 and type 2 diabetes mellitus. Analyzing data from the Victor Babes Hospital for Infectious Diseases and Pulmonology over three years, the study identifies that T2DM patients faced notably harsher outcomes from COVID-19. These individuals experienced higher rates of severe cases, increased mortality, and a greater likelihood of requiring intensive care and mechanical ventilation compared to their T1D counterparts. Additionally, T2D patients had longer hospital stays and presented with higher levels of hyperglycemia upon admission. These findings underscore the importance of targeted strategies in managing T2D patients with COVID-19, considering their elevated risk profile. The study highlights the evolving nature of the pandemic’s impact on different diabetic subtypes, emphasizing the need for continuous monitoring and tailored healthcare approaches for patients with diabetes.

## Figures and Tables

**Table 1:** Background characteristics of COVID-19 patients with and without diabetes mellitus.

Variables	No Diabetes (*n* = 200)	T1D (*n* = 62)	T2D (*n* = 224)	*p*-Value	*p*-Value *
Age, years (mean ± SD)	55.3 ± 12.5	53.6 ± 11.7	56.4 ± 13.1	0.281	0.129
Gender, men	118 (59.0%)	35 (56.5%)	121 (54.0%)	0.586	0.733
BMI (mean ± SD)	25.8 ± 4.1	24.7 ± 5.0	26.0 ± 6.3	0.234	0.135
Currently smoking	58 (29.0%)	20 (32.3%)	68 (30.4%)	0.878	0.774
Alcohol use (frequent)	19 (9.5%)	4 (6.5%)	12 (5.4%)	0.249	0.739
Place of origin (urban)	126 (63.0%)	40 (64.5%)	144 (64.3%)	0.955	0.973
COVID-19 vaccinated	52 (26.0%)	20 (32.3%)	68 (30.4%)	0.498	0.774
CCI > 2	46 (23.0%)	17 (27.4%)	56 (25.0%)	0.756	0.698
COVID-19 severity				<0.001	0.498
Mild	99 (49.5%)	22 (35.5%)	68 (30.4%)		
Moderate	71 (35.5%)	24 (38.7%)	81 (36.2%)		
Severe	30 (15.0%)	16 (25.8%)	75 (33.5%)		
Duration of diabetes diagnosis, years (mean ± SD)	-	10.9 ± 4.6	9.6 ± 5.1	-	0.071
Diabetes-related complications					
Cardiovascular disease	-	26 (41.9%)	108 (48.2%)	-	0.380
Neuropathy	-	19 (30.6%)	81 (36.2%)	-	0.420
Kidney disease	-	13 (21.0%)	75 (33.5%)	-	0.058
Others	-	9 (14.5%)	36 (16.1%)	-	0.766
Hyperglycemia at admission	-	20 (32.3%)	116 (51.8%)	-	0.006
Insulin use	-	62 (100%)	38 (17.0%)	-	<0.001
Dialysis users	-	5 (8.1%)	27 (12.1%)	-	0.377
ICU admissions	34 (17.0%)	14 (22.6%)	71 (31.7%)	0.002	0.164
Mechanical ventilation	36 (18.0%)	18 (29.0%)	79 (35.3%)	<0.001	0.358
Mortality	7 (3.5%)	5 (8.1%)	26 (11.6%)	0.008	0.427

*—Comparison between T1D and T2D groups; SD—standard deviation; BMI—body mass index; T1D—type 1 diabetes; T2D—type 2 diabetes; CCI—Charlson Comorbidity Index; ICU—intensive care unit.

**Table 2:** Laboratory findings at admission.

Variables	Normal Range	No Diabetes (*n* = 200)	T1D (*n* = 62)	T2D (*n* = 224)	*p*-Value	*p*-Value *
Blood Glucose (mg/dL)	70–99	95.1 ± 15.2	164.8 ± 39.6	178.3 ± 34.7	<0.001	0.009
HbA1c (%)	4.0–5.6	5.6 ± 0.5	7.7 ± 1.1	8.1 ± 1.4	<0.001	0.038
C-Reactive Protein (mg/L)	<5.0	15.2 ± 8.9	27.4 ± 13.8	38.2 ± 14.6	<0.001	<0.001
D-Dimer (μg/mL)	<0.5	1.6 ± 1.2	2.8 ± 1.3	2.9 ± 2.0	<0.001	0.798
Oxygen Saturation (%)	95–100	93.9 ± 10.8	91.7 ± 12.9	90.8 ± 13.7	0.037	0.643
Creatinine (mg/dL)	0.7–1.2	0.9 ± 0.4	1.2 ± 0.6	1.3 ± 0.9	<0.001	0.410
Lymphocyte Count (×10^9^/L)	1.0–3.0	3.4 ± 2.5	3.2 ± 2.6	3.1 ± 2.4	0.455	0.775
WBC Count (×10^9^/L)	4.5–11.0	9.0 ± 6.6	10.5 ± 4.8	10.2 ± 5.0	0.052	0.673

*—Comparison between T1D and T2D groups; T1D—type 1 diabetes; T2D—type 2 diabetes; WBC—white blood cell; HbA1c—Hemoglobin A1c.

**Table 3:** Patient outcomes.

Variables	No Diabetes (*n* = 200)	T1D (*n* = 62)	T2D (*n* = 224)	*p*-Value	*p*-Value *
Length of hospital stay (days)	7.2 ± 6.6	9.1 ± 5.8	11.6 ± 7.0	<0.001	0.010
Received antiviral treatment (%)	156 (78.0%)	51 (82.3%)	192 (85.7%)	0.117	0.501
Received steroids (%)	121 (60.5%)	43 (69.4%)	167 (74.6%)	0.008	0.412
Non-invasive ventilation (%)	62 (31.0%)	28 (45.2%)	119 (53.1%)	<0.001	0.266
Discharge status (recovered) (%)	193 (96.5%)	57 (91.9%)	198 (88.4%)	0.008	0.427

*—Comparison between T1D and T2D groups; T1D—type 1 diabetes mellitus; T2D—type 2 diabetes mellitus.

**Table 4:** Evolution of COVID-19 outcomes over time.

Year	COVID-19 Severity		Length of Hospital Stay		ICU Admissions		Mortality	
	T1D	T2D	T1D	T2D	T1D	T2D	T1D	T2D
2020	Moderate: 30.6%, Severe: 14.5%	Moderate: 33.9%, Severe: 17.0%	9.0 ± 5.8 *	10.8 ± 6.1 *	11.3%	15.2%	4.0%	5.6%
2021	Moderate: 35.5%, Severe: 20.3%	Moderate: 40.2%, Severe: 25.0%	9.9 ± 6.3 *	12.4 ± 6.9 *	19.4%	26.3%	7.7%	11.2%
2022	Moderate: 32.3%, Severe: 16.1%	Moderate: 36.6%, Severe: 21.9%	7.8 ± 4.9 *	9.6 ± 5.6 *	14.5%	20.5%	6.1%	9.4%

*—Statistically significant differences between T1D and T2D groups; T1D—type 1 diabetes; T2D—type 2 diabetes.

**Table 5:** Correlation matrix of COVID-19 outcomes and characteristics.

Variables	Age	BMI	COVID-19 Severity	Hyperglycemia at Admission	ICU Admissions	Mechanical Ventilation	Mortality	Diabetes Type
Age	1	−0.315	0.124	−0.206	0.289 *	−0.148	0.032	−0.178
BMI	−0.315	1	−0.473 *	0.217	−0.356	0.439 *	−0.112	0.264
COVID-19 Severity	0.124	−0.473 *	1	−0.528 *	0.603 *	−0.287	0.456 *	−0.219
Hyperglycemia at Admission	−0.206	0.217	−0.528 *	1	−0.192	0.348	−0.414 *	0.327
ICU Admissions	0.289 *	−0.356	0.603 *	−0.192	1	−0.311	0.279	−0.243
Mechanical Ventilation	−0.148	0.439 *	−0.287	0.348	−0.311	1	−0.253	0.197
Mortality	0.032	−0.112	0.456 *	−0.414 *	0.279	−0.253	1	−0.287
Diabetes Type	−0.178	0.264	−0.219	0.327	−0.243	0.197	−0.287	1

*—Statistically significant differences; ICU—intensive care unit.

**Table 6:** COVID-19 outcomes by diabetes status.

Variables	No Diabetes (*n* = 200)	T1D (n = 62)	T2D (*n* = 224)	OR (T2D vs. None) (95% CI)	OR (T2D vs. T1D) (95% CI)
ICU admission risk	17.00%	22.60%	31.70%	2.24 (1.40–3.57) *	1.59 (1.09–2.83) *
Mechanical Ventilation Risk	18.00%	29.00%	35.30%	2.46 (1.55–3.90) *	1.37 (0.81–2.30)
Mortality	3.50%	8.10%	11.60%	3.60 (1.08–8.20) *	1.48 (0.58–3.76)
Hospitalization Duration (Days)	7.2 ± 6.6	9.1 ± 5.8	11.6 ± 7.0	1.61 (1.25–2.08) *	1.28 (1.07–1.68) *

*—Statistically significant differences (*p*-value < 0.05); T1D—type 1 diabetes; T2D—type 2 diabetes; ICU—intensive care unit; odds ratios (OR) with 95% confidence intervals (CI) compare type 1 and type 2 diabetes to the control group (no diabetes). The logistic regression model was controlled for age, gender, BMI, and comorbidities. Model fit statistics: Hosmer–Lemeshow test *p* = 0.320, indicating a good fit.

## Data Availability

The data presented in this study are available on request from the corresponding author.
